# Targeting the Hippo Pathway in Breast Cancer: A Proteomic Analysis of Yes-Associated Protein Inhibition

**DOI:** 10.3390/ijms26093943

**Published:** 2025-04-22

**Authors:** Sevinc Yanar, Merve Gulsen Bal Albayrak, Tuğcan Korak, Asuman Deveci Ozkan, Sevil Arabacı Tamer, Murat Kasap

**Affiliations:** 1 Department of Histology and Embryology, Faculty of Medicine, Sakarya University, 54187 Sakarya, Turkey; 2 Department of Medical Biology, Faculty of Medicine, Kocaeli University, 41380 Kocaeli, Turkey; merve.bal@kocaeli.edu.tr (M.G.B.A.); tugcan.korak@kocaeli.edu.tr (T.K.); mkasap@kocaeli.edu.tr (M.K.); 3 Department of Medical Biology, Faculty of Medicine, Sakarya University, 54187 Sakarya, Turkey; deveci@sakarya.edu.tr; 4 Department of Physiology, Faculty of Medicine, Sakarya University, 54187 Sakarya, Turkey; sarabaci@sakarya.edu.tr

**Keywords:** hippo pathway, breast cancer, yes-associated protein (YAP), CA3 (CIL56), proteomics, apoptosis, autophagy, DNA repair, metabolic reprogramming

## Abstract

The dysregulation of the Hippo signaling pathway leads to the aberrant activation of oncogenic YAP and TAZ, driving tumor progression. In breast cancer, this disruption promotes proliferation and metastasis. This study investigates the effects of CA3, a selective YAP inhibitor, on the proteome of triple-negative breast cancer MDA-MB-231 and luminal-A-like MCF7 cells. Proteomic changes were analyzed via nano-LC-MS/MS, while cytotoxicity, apoptosis, and autophagy were assessed through WST-1 assays, flow cytometry, and Western blot analyses. Bioinformatics tools were employed to identify enriched pathways. MDA-MB-231 cells exhibited an increased expression of DNA repair proteins (*p* < 0.05), indicating a compensatory response to maintain genomic stability. In contrast, MCF7 cells showed a downregulation of DNA repair factors (*p* < 0.005). Additionally, metabolic reprogramming was apparent in MCF7 cells (*p* < 0.001). Apoptosis assays revealed a rise in cell death, while cell cycle analysis indicated pronounced G1-phase arrest in MDA-MB-231 cells (*p* < 0.01). Moreover, autophagic suppression was particularly evident in MCF7 cells. This study, for the first time, provides evidence that breast cancer subtypes exhibit distinct dependencies on YAP-driven pathways, revealing potential therapeutic vulnerabilities. Targeting Hippo signaling alongside DNA repair in triple-negative breast cancer or combining YAP inhibition with metabolic blockade in luminal breast cancer holds significant potential to enhance treatment efficacy.

## 1. Introduction

Breast cancer is one of the most common malignant tumors globally and remains the second leading cause of cancer-associated deaths in women [1]. Marked by heterogeneity and diverse histopathological subtypes, it displays distinct gene expression profiles [2]. As a complex and widespread malignancy, breast cancer continues to impose a significant burden on global health. Understanding the molecular mechanisms and cellular pathways underlying its initiation and progression is crucial for the development of targeted therapeutic approaches.

Among the complex signaling networks implicated in cancer, the Hippo signaling pathway has been identified as a crucial regulator of cellular homeostasis and organ development [3]. First identified in *Drosophila melanogaster* [4], this evolutionarily conserved pathway governs tissue growth and organ size across various species, including humans. The core components of the Hippo pathway include the mammalian sterile 20-like kinases 1 and 2 (MST1/2), large tumor suppressors 1 and 2 (LATS1/2), and the adaptor proteins Salvador homolog 1 (SAV1) and MOB kinase activator 1A/B (MOB1A/B) [3]. When activated, the pathway induces the phosphorylation of LATS1/2, which subsequently phosphorylates the transcriptional coactivators Yes-associated protein (YAP) and transcriptional coactivator with PDZ-binding motif (TAZ). This phosphorylation process retains YAP and TAZ in the cytoplasm, preventing their nuclear translocation and blocking their transcriptional activity [5]. When the Hippo pathway is inactive due to factors like changes in cell density or loss of cell contact, YAP and TAZ move into the nucleus. There, they bind to TEAD transcription factors, triggering the activation of genes that promote cell growth, survival, and specialization [6,7].

The Hippo pathway functions as a tumor suppressor by limiting YAP/TAZ activity, thus preventing uncontrolled cell growth and promoting apoptosis to maintain tissue homeostasis [8]. However, when this pathway is disrupted, leading to the aberrant activation of YAP/TAZ, it has been associated with several cancers, including non-small cell lung cancer, gastric cancer, hepatocellular carcinoma, renal cell carcinoma, colorectal cancer, leukemia, and breast cancer. The overexpression of YAP/TAZ has been linked to poor prognosis, as it accelerates tumor growth, metastasis, and resistance to therapy [9,10].

In breast cancer, the activation of YAP and TAZ has been found to drive tumor growth, metastasis, drug resistance, tumor microenvironment modulation, and angiogenesis [11,12]. Their interaction with TEAD transcription factors regulates crucial processes such as proliferation, survival, and therapy resistance, further highlighting the pathway’s role in cancer progression [11,12].

Given the significance of the Hippo pathway in breast cancer, significant efforts have been made to develop therapeutic strategies targeting this pathway. Current approaches focus on activating MST and LATS kinases, inhibiting YAP/TAZ regulators, and disrupting YAP/TAZ-TEAD interactions. Several small molecules, including verteporfin (VP), Super-TDU, CPD3.1, and CA3, have shown potential in suppressing YAP/TAZ activity and reducing tumor growth [13,14]. Among these, CA3 has exhibited strong and specific YAP inhibition, significantly reducing tumor size in preclinical models [15]. It has been shown to inhibit YAP activity by disrupting the YAP–TEAD interaction and reducing YAP levels without altering upstream Hippo components [16]. The molecule has demonstrated strong antitumor effects in multiple cancer models, including esophageal adenocarcinoma [16], mesothelioma [17], hepatocellular carcinoma [18], and cholangiocarcinoma [19], particularly in tumors enriched with YAP-driven cancer stem cell populations. In esophageal adenocarcinoma, CA3 significantly suppressed the growth of radiation-resistant, cancer stem cell-like populations and enhanced the cytotoxic effects of 5-fluorouracil (5-FU), highlighting its potential in overcoming therapy resistance [16]. In mesothelioma and liver cancer models, CA3 reduced tumor spheroid formation, induced apoptosis, and synergized with targeted therapies [17,18]. These studies highlight CA3’s potential as a selective and effective YAP inhibitor, supporting its relevance in targeting YAP/TAZ-driven oncogenesis. However, the precise mechanisms governing YAP/TAZ function and their role in breast cancer progression remain incompletely understood.

Although there is increasing evidence linking Hippo pathway dysregulation to breast cancer, there is limited research examining its impact on the breast cancer proteome. Since changes at the protein level can provide important information about tumor biology and potential therapeutic targets, investigating the regulatory effects of Hippo signaling on the breast cancer proteome is essential. This study investigates how the modulation of the Hippo pathway through the YAP inhibitor CA3 impacts the breast cancer cell proteome, shedding light on crucial molecular mechanisms that drive tumor progression. By uncovering these mechanisms, we aim to identify novel therapeutic targets that could pave the way for more effective breast cancer treatments.

## 2. Results

### 2.1. CA3-Induced Cytotoxicity and Selectivity

**Figure 1:**
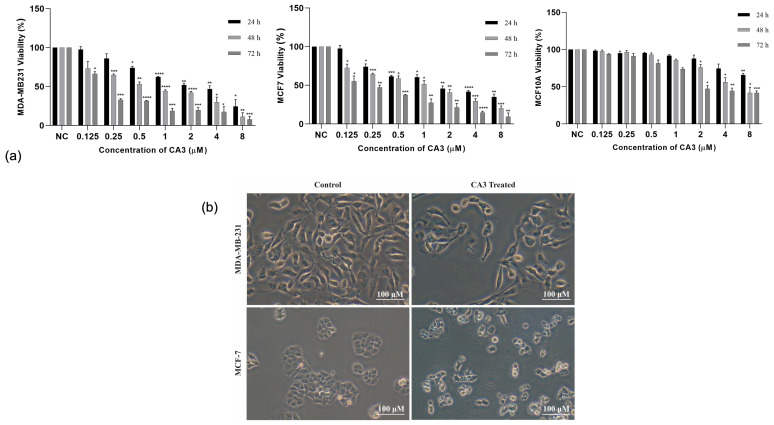
(**a**) The effects of CA3 on the viability of MDA-MB231, MCF7, and MCF10A cells. (NC: negative control, * *p* < 0.05, ** *p* < 0.005, *** *p* < 0.0005, **** *p* < 0.0001). (**b**) Representative optical microscopy images showing morphological changes in MDA-MB-231 and MCF7 cells after treatment with CA3 at their respective IC_50_ concentrations, compared to control.

CA3 treatment led to a concentration- and time-dependent decrease in the viability of both MDA-MB231 and MCF7 breast cancer cells. Notably, MDA-MB-231 cells exhibited a greater sensitivity to CA3, with significant reductions in viability occurring at lower concentrations compared to MCF7 cells (Figure 1a). A significant dose-dependent reduction in viability (*p* < 0.05) was observed at 0.5 μM and higher, with more strong effects seen after 48 and 72 h of treatment. Likewise, in MCF7 cells, CA3 led to a substantial decrease in cell viability, particularly at concentrations of 2 μM and above (*p* < 0.05). To evaluate the selectivity of CA3, its effects were tested on the non-tumorigenic mammary epithelial cell line MCF10A using the same concentration range. Statistically significant reductions in viability were only observed at the highest concentrations and after prolonged exposure, suggesting that CA3 preferentially targets cancer cells while sparing normal mammary epithelial cells (Figure 1a).

The half-maximal inhibitory concentration (IC_50_) values for CA3 after 48 h of treatment were determined using a linear regression model. The IC_50_ values were calculated as 0.5 μM for MDA-MB-231 cells and 1 μM for MCF7 cells. To visually assess the morphological effects of CA3 at these IC_50_ concentrations, optical microscopy analysis was performed after 48 h of treatment. CA3 induced marked morphological alterations in both MDA-MB-231 and MCF7 cells, including cell shrinkage and loss of adhesion, compared to untreated controls (Figure 1b).

### 2.2. YAP Inhibition by CA3 in Breast Cancer Cells

**Figure 2:**
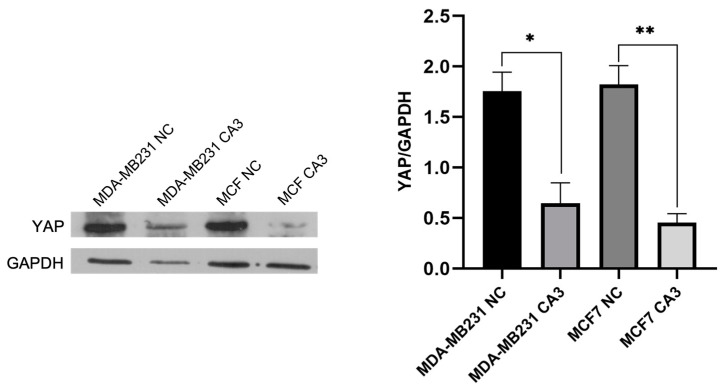
Representative Western blot images and relative expression levels of YAP in MDA-MB231 and MCF7 cells after 48 h of CA3 treatment. CA3 was applied at its IC_50_ concentration: 0.5 μM for MDA-MB-231 and 1 μM for MCF7 (NC: negative control, * *p* < 0.0005, ** *p* < 0.0001).

To confirm the inhibitory effect of CA3 on YAP expression before conducting proteomic analysis, Western blot analysis was performed in MDA-MB231 and MCF7 cells. Following CA3 treatment at their respective IC_50_ concentrations, YAP levels were significantly reduced in both cell lines compared to the control group (Figure 2). The reduction was highly significant, with MDA-MB-231 cells showing *p* < 0.0005 and MCF7 cells exhibiting *p* < 0.0001. These results confirmed that CA3 effectively suppressed YAP expression in both cancer cell lines and supported its role in modulating the Hippo signaling pathway.

### 2.3. Label-Free Quantification and Protein Identification

Proteins identified in CA3-treated MDA-MB231 and MCF7 cells were analyzed using a label-free quantification approach. The initial analysis detected 2196 proteins in MDA-MB-231 cells and 2198 proteins in MCF7. Filtering criteria were applied to enhance the reliability and biological significance of the data. Proteins exhibiting high false discovery rate confidence and an abundance ratio above two were selected as priorities for further analysis. Following these criteria, the number of DEPs was obtained as 73 in MDA-MB-231 cells, including 28 upregulated and 45 downregulated proteins, and 425 in MCF7 cells, including 134 upregulated and 291 downregulated proteins. The overlap of differentially expressed proteins between groups in the two cell lines is shown in Venn diagrams (Supplementary Figure S1).

### 2.4. Bioinformatics Analysis and Validation of Differentially Expressed Proteins

**Figure 3:**
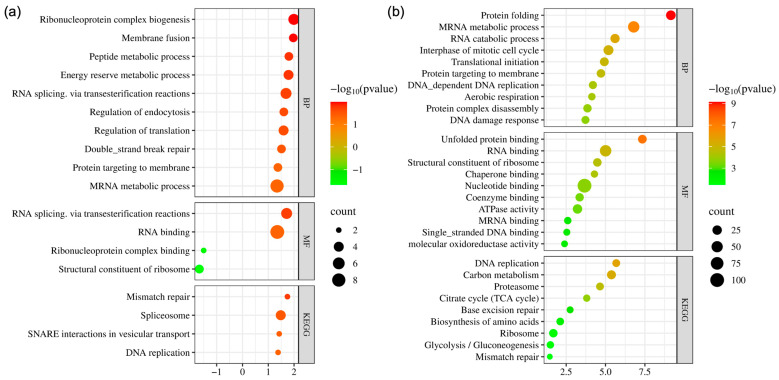
Pathway enrichment analysis visualized using bubble plots for CA3-treated (**a**) MDA-MB231 and (**b**) MCF7 cells based on data from Gene Ontology (GO); BP: biological function, MF: molecular function, KEGG: Kyoto Encyclopedia of Genes and Genomes.

**Figure 4:**
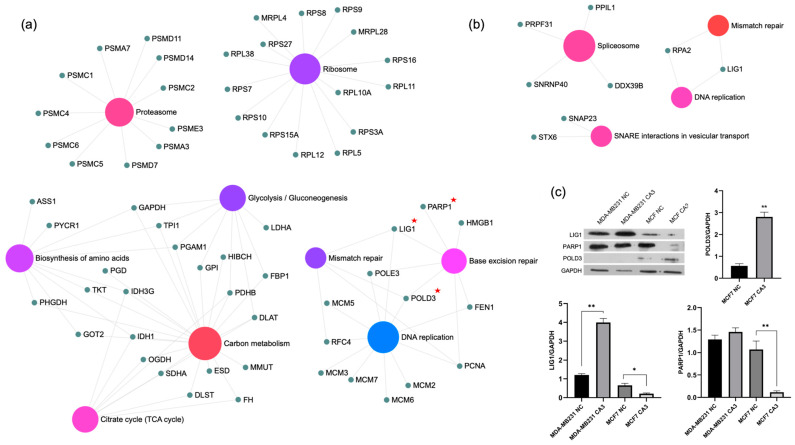
KEGG pathway enrichment analysis using ExpressAnalyst for MDA-MB-231 (**a**) and MCF7 (**b**) cells. Each colored node represents a distinct functional pathway category (e.g., proteasome, ribosome, DNA repair, metabolism), as labeled directly on the graph. Small gray nodes indicate individual proteins associated with these pathways. Proteins marked with a red star (LIG1, POLD3, and PARP1) are selected for validation. (**c**) Western blot analysis of these proteins, with corresponding quantifications presented in bar graphs (NC: negative control, * *p* < 0.0005, ** *p* < 0.0001).

Bioinformatics analysis of DEPs in MDA-MB-231 cells revealed significant enrichment in biological processes related to RNA processing and DNA repair (Figure 3a). “Ribonucleoprotein complex biogenesis”, “Membrane fusion”, “Peptide metabolic process”, and “RNA splicing” were notably enriched, reflecting active RNA metabolism and regulation. Double-strand break repair was also highly enriched, indicating the critical involvement of DNA repair mechanisms. Enriched molecular functions included “RNA splicing”, “RNA binding”, and roles in ribosome biogenesis, reflecting the active involvement of these proteins in RNA regulation and protein synthesis. Similarly, KEGG pathway analysis highlighted pathways related to DNA repair and RNA processing, including “Mismatch repair”, “Spliceosome”, and “DNA replication”. A detailed view of the KEGG pathway enrichment is presented in Figure 4a. DEPs associated with mismatch repair and DNA replication, namely LIG1 and RPA2, were found to be upregulated in these cells.

For MCF7 cells, bioinformatics analysis revealed significant changes in pathways related to DNA repair, RNA metabolism, and energy metabolism (Figure 3b). Among biological processes, “Protein folding”, “mRNA metabolic process”, and “RNA catabolic process” were notably enriched in addition to “Aerobic respiration”, “DNA replication”, and “DNA damage response”. The molecular function analysis further emphasized the importance of RNA binding and ribosomal activity, including enrichments related to “Unfolded protein binding”, “Chaperone binding”, and “ATPase activity”. Additional functions such as “RNA binding” and “Single-stranded DNA binding” pointed to the active regulation of RNA and DNA processing mechanisms. KEGG pathway analysis identified significant enrichment in pathways related to DNA repair and metabolism, including “DNA replication”, “Base excision repair”, and “Mismatch repair”. DEPs associated with DNA replication and repair mechanisms included POLD3, PCNA, LIG1, RFC4, HMGB1, POLE3, FEN1, and PARP1. Despite a significant increase in POLD3 expression, all other proteins were found to be downregulated. Enriched metabolic pathways, such as “Carbon metabolism”, “Citrate cycle (TCA cycle)”, and “Glycolysis/Gluconeogenesis”, indicated an adaptive metabolic response to treatment. Additionally, “Proteasome” and “Biosynthesis of amino acids” highlighted the importance of protein degradation and synthesis processes (Figure 4b).

To further investigate and confirm the effects of CA3 on DNA replication and repair mechanisms, Western blot analysis was performed for selected DEPs, including LIG1 (DNA ligase I), PARP1 (Poly [ADP-ribose] polymerase 1), and POLD3 (DNA polymerase delta subunit 3) (Figure 4c). CA3 treatment resulted in a significant upregulation of LIG1 in MDA-MB-231 cells (*p* < 0.0001), whereas a notable downregulation was observed in MCF7 cells (*p* < 0.0005). PARP1 level slightly increased in MDA-MB-231 cells but exhibited a marked reduction in MCF7 cells (*p* < 0.0001), aligning with the proteomics data. POLD3, detected exclusively in MCF7 cells based on proteomic analysis, was strongly upregulated following CA3 exposure (*p* < 0.0001). Collectively, these findings further validated the proteomic results, highlighting the role of YAP modulation in regulating key proteins involved in DNA replication and repair.

### 2.5. Induction of Apoptosis and Cell Cycle Modulation

**Figure 5:**
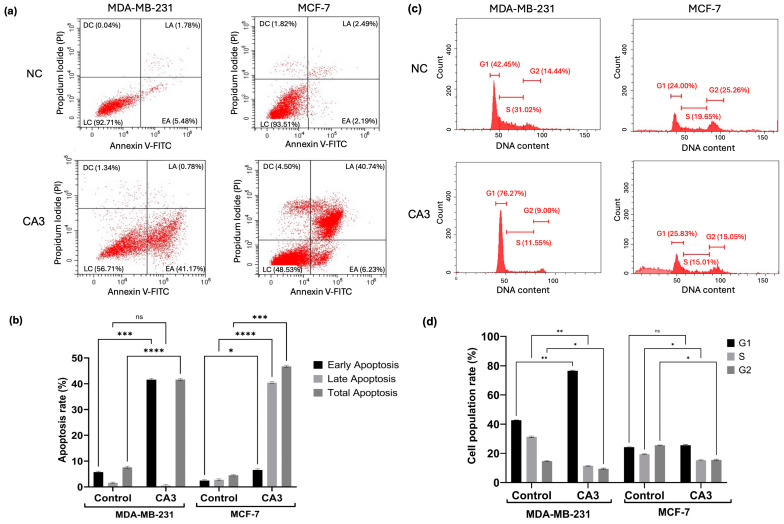
The effects of CA3 treatment for 48 h on apoptosis and cell cycle distribution in MDA-MB-231 and MCF7 cells. (**a**) Representative flow cytometry dot plots of Annexin V/PI staining. The percentage of cells in each quadrant (LC: live cell, EA: early apoptosis, LA: late apoptosis, and DC: death cell) is indicated. (**b**) Statistical analysis of early, late, and total apoptosis rates based on Annexin V/PI staining from three independent experiments. (**c**) Representative flow cytometric histograms showing DNA content profiles of cells stained with PI for cell cycle analysis. The proportions of cells in G1, S, and G2/M phases are indicated. (**d**) Statistical analysis of cell cycle distribution in G1, S, and G2/M phases. The percentages were calculated based on PI-stained DNA content using flow cytometry. Data represent mean values from three independent experiments (ns: not significant,* *p* < 0.05, ** *p* < 0.01, *** *p* < 0.001, **** *p* < 0.0001).

To determine the apoptotic effects of YAP inhibition in MDA-MB231 and MCF7 cells, Annexin V/PI staining was performed and analyzed via flow cytometry (Figure 5a). Quantitative analysis revealed a significant increase in early and late apoptotic populations following CA3 treatment in both cell lines compared to their respective controls (Figure 5b). MDA-MB-231 cells exhibited a significant increase in apoptosis, with a notable elevation in early apoptotic cells (*p* < 0.001) and total apoptosis (*p* < 0.0001). In MCF-7 cells, CA3 induced a marked increase in early (*p* < 0.05) and late apoptosis (*p* < 0.0001), leading to a substantial rise in total apoptotic cell percentage (*p* < 0.001). In MDA-MB-231 cells, CA3 induced a strong G1-phase arrest

CA3 treatment also had distinct effects on the cell cycle progression in two cell lines (Figure 5c). In MDA-MB-231 cells, CA3 induced strong G1-phase arrest, as evidenced by a significant increase in the G1 population (*p* < 0.01) and a corresponding decrease in the S phase (*p* < 0.01) and G2 phase (*p* < 0.05). In contrast, MCF-7 cells revealed no significant changes in the G1 phase, whereas a significant decrease in the S phase and G2 phase was detected (*p* < 0.05). (Figure 5d).

### 2.6. Autophagic Markers Following YAP Inhibition

**Figure 6:**
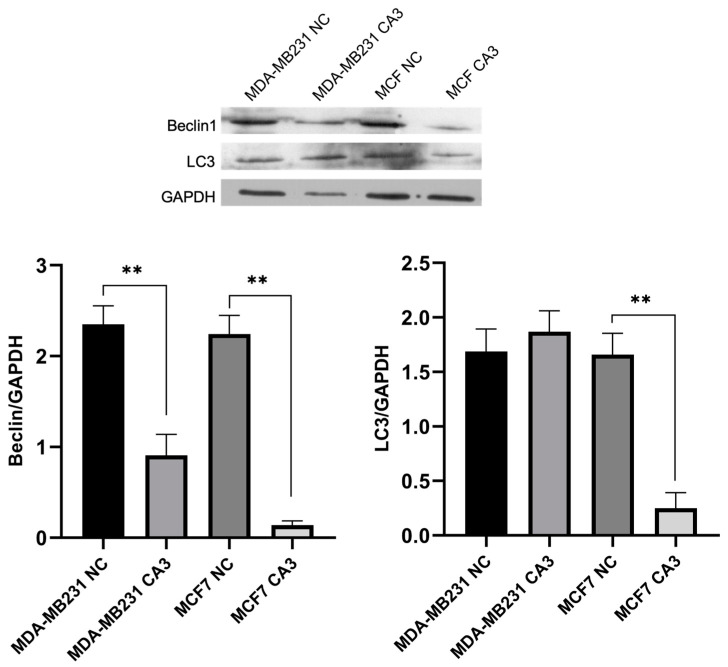
Representative Western blot images and relative expression levels of Beclin1 and LC3 protein expression in CA-3-treated MDA-MB231 and MCF7 cells (NC: negative control, ** *p* < 0.0001).

To examine the impact of YAP inhibition on autophagy, Beclin1 and LC3 levels were analyzed by Western blot, followed by visualization of autophagic vesicle formation. Quantitative analysis revealed a significant reduction in Beclin1 expression after treatment in both cell lines (*p* < 0.0001). In MDA-MB-231 cells, LC3 levels remained relatively unchanged following CA3 treatment, whereas MCF7 cells exhibited a significant reduction in LC3 expression (*p* < 0.0001) (Figure 6).

## 3. Discussion

The large difference in the number of DEPs between MCF7 and MDA-MB-231 after YAP inhibition may be due to the distinct molecular characteristics of these cells and their dependence on the Hippo signaling pathway. MCF7 cells are characterized as estrogen receptor-positive and belong to the luminal A subtype, which typically exhibits a less aggressive phenotype. In contrast, MDA-MB-231 cells are classified as triple-negative breast cancer (TNBC) and are associated with a more invasive and metastatic behavior [20]. Studies have shown that YAP expression is higher in luminal breast cancer cells, compared to TNBC subtypes [21]. Similarly, in our study, Western blot analysis revealed higher baseline YAP levels in MCF7 cells compared to MDA-MB-231 cells. Furthermore, following CA3 treatment, a significantly greater reduction in YAP1 expression was observed in MCF7 cells. These all may suggest that MCF7 cells may be more dependent on YAP activity for survival and proliferation.

MDA-MB-231 cells exhibited significant enrichment in RNA processing and DNA repair pathways, with a considerable upregulation of mismatch repair and replication-associated proteins LIG1 and RPA2. This increased activity suggests TNBC cells rely on efficient DNA repair mechanisms to provide genomic instability. For instance, Andrade et al. demonstrated that YAP inhibition impairs DNA double-strand break repair in TNBC cells, rendering them more susceptible to DNA damage-inducing therapies [22]. Similarly, elevated RAD18 expression is associated with advanced TNBC and poorer prognosis, suggesting that increased DNA repair activity contributes to the aggressiveness of these cancers [23].

LIG1 plays a pivotal role in DNA replication, base excision repair (BER), and mismatch repair. Increased LIG1 expression in MDA-MB-231 cells suggests an adaptive mechanism to counteract the genomic instability characteristic of TNBCs [24]. RPA2, a component of the replication protein A complex, is a key player in DNA replication and repair, and facilitates the recruitment of proteins involved in nucleotide excision repair and homologous recombination [25]. The increased expression of these proteins may suggest that MDA-MB-231 cells rely on intact DNA repair pathways to counteract YAP inhibition. Notably, the upregulation of LIG1 was validated by Western blot analysis, confirming the proteomic findings.

Moreover, the observed enrichment in ribonucleoprotein complex biogenesis and RNA splicing underscores the role of YAP signaling in regulating RNA metabolism in aggressive breast cancer phenotypes. YAP has been shown to promote global mRNA translation, thereby fueling oncogenic growth [26]. Additionally, YAP/TAZ have been found to enhance processing body formation, which contributes to tumorigenesis [27]. These findings suggest that MDA-MB-231 cells may benefit from YAP-mediated modulation of RNA processing networks to promote their aggressive behavior.

The bioinformatics analysis of MCF7 cells revealed significant alterations in DNA repair, RNA metabolism, and energy homeostasis. One of the most notable findings in MCF7 cells was the significant upregulation of POLD3, despite the downregulation of other essential DNA repair proteins, including PCNA, LIG1, RFC4, HMGB1, POLE3, FEN1, and PARP1. POLD3 plays a critical role in lagging-strand DNA synthesis, mismatch repair, and homologous recombination, and its overexpression has been associated with enhanced replication fidelity and resistance to DNA-damaging agents [28,29]. The significant increase in POLD3 may indicate an attempt by MCF7 cells to maintain replication stability under CA3-induced stress.

However, the downregulation of other important repair proteins suggests that despite POLD3 upregulation, MCF7 cells may have an impaired overall DNA repair capacity. The downregulation of PCNA, a major player in replication-associated DNA repair, is often associated with reduced DNA damage tolerance and increased sensitivity to replication stress [30]. Similarly, LIG1 deficiency can lead to incomplete DNA repair, resulting in increased DNA strand breaks and genomic instability [31,32]. Another critical downregulated protein was PARP1, a central enzyme in single-strand break repair and a key factor in the BER pathway. The suppression of PARP1 expression suggests a reduction in BER activity, making MCF7 cells potentially more susceptible to DNA damage accumulation [33]. Additionally, the suppression of RFC4 and POLE3, both involved in DNA replication and repair, suggests an overall reduction in the efficiency of DNA replication and repair processes. Notably, the differential expression patterns of LIG1, PARP1, and POLD3 in MCF7 cells were further validated through Western blot analysis. The findings confirmed the proteomic results by demonstrating significant differences between treated and control groups, highlighting their regulatory roles in DNA repair mechanisms in response to YAP inhibition.

MCF7 cells exhibited significant changes in energy metabolism pathways, indicating adaptive metabolic reprogramming. This adaptation allows cancer cells to maintain ATP production and essential biosynthetic processes under therapeutic stress [34,35]. Notably, the enhancement of TCA cycle-related pathways suggests a potential metabolic flexibility or a shift toward oxidative phosphorylation as a survival strategy. This metabolic flexibility has been shown to enable cancer cells to switch between glycolysis and oxidative phosphorylation in response to environmental and therapeutic pressures [36]. 

The enrichment of RNA metabolism and protein homeostasis observed in MCF cells implies post-transcriptional regulation of key genes involved in DNA repair and energy metabolism, potentially influencing the differential expression of factors such as POLD3, PCNA, and PARP1. Meanwhile, the upregulation of protein folding and chaperone activity highlights an effort to stabilize repair proteins and mitigate proteotoxic stress. The interaction between RNA regulation and metabolic pathways may be due to enhanced RNA processing, which increases the metabolic flexibility required for survival under stress.

Differences in DNA repair and metabolism regulation led us to examine survival pathways like apoptosis and autophagy. Apoptosis analysis helps determine if proteomic and molecular changes increase cell death, while autophagy can either support survival under stress or cause cell death if overactivated. Linking these pathways to proteomic data helps reveal whether cells are driven toward apoptosis or using autophagy as a defense mechanism. The dysregulation of the Hippo pathway, particularly the overactivation of YAP/TAZ, has been implicated in various cancers, leading to uncontrolled cell proliferation and resistance to apoptosis [37]. Conversely, the inhibition of YAP/TAZ has been reported to enhance apoptotic processes in cancer cells [38,39]. In the present study, there was a significant increase in early and late apoptotic populations in CA3-treated cells, supporting that YAP plays an important role in promoting cell survival in these breast cancer cells.

In MDA-MB-231 cells, the pronounced G1-phase arrest corresponds with the upregulation of DNA repair proteins LIG1 and RPA2. This arrest likely serves as a cellular response to facilitate DNA repair before cell cycle progression, highlighting the dependency of TNBC cells on efficient DNA repair mechanisms to maintain genomic stability [22]. Conversely, in MCF7 cells, the absence of significant G1-phase arrest and reductions in S and G2 phases could be related to the downregulation of key DNA repair proteins such as PCNA, LIG1, and PARP1. This situation points to the compromised DNA repair capacity, potentially leading to replication stress and increased apoptosis.

Recent studies have also highlighted the interplay between the Hippo pathway and autophagy [40,41]. While YAP/TAZ activation generally promotes autophagy [42,43], facilitating cancer cell survival under stress conditions, there are context-dependent variations. For instance, in certain scenarios, YAP has been shown to inhibit autophagy, thereby influencing cancer progression differently [44]. Western blot analyses showed that YAP inhibition resulted in a significant reduction in Beclin1 expression in both cell lines. Beclin1 is a crucial initiator of autophagy, and its downregulation suggests a suppression of autophagic processes [45]. Interestingly, while MCF7 cells exhibited a significant decrease in LC3 expression, a marker of autophagosome formation [46], indicating reduced autophagic activity, LC3 levels in MDA-MB-231 cells remained relatively unchanged. These unaltered LC3 levels could suggest alternative autophagy regulatory mechanisms independent of YAP or a basal level of autophagy that persists despite Beclin1 reduction.

The observed reduction in autophagic activity in MCF7 cells seems to support the proteomic data showing an extensive downregulation of key DNA repair proteins. The diminished autophagic response may limit the cells′ ability to manage accumulating DNA damage and proteotoxic stress effectively, exacerbating their susceptibility to apoptosis. In contrast, the relative stability of LC3 expression in MDA-MB-231 cells, despite decreased Beclin1 levels, correlates with the cells′ proteomic profile showing enhanced expression of DNA repair proteins. This stable autophagic activity may support DNA repair processes, helping these cells better survive the stress caused by YAP inhibition.

This study provides a comprehensive proteomic and functional analysis of YAP inhibition in TNBC using CA3, a selective and potent inhibitor. While our findings highlight important changes in cellular pathways following CA3 treatment, future studies could benefit from comparing the effects of additional YAP inhibitors with varying mechanisms of action, such as verteporfin or Super-TDU. Such comparative analyses would help to further validate the generalizability of the observed responses and broaden our understanding of how the Hippo pathway can be targeted in breast cancer.

## 4. Materials and Methods

### 4.1. Cytotoxicity Assessment of CA3 in Breast Cancer Cells

The human breast cancer cell lines MDA-MB231 and MCF7, along with the normal breast cell line MCF10A, were cultured in DMEM medium (Thermo Fisher Scientific, Waltham, MA, USA) supplemented with high glucose, fetal bovine serum (FBS, 10%), 100 μg/mL streptomycin, and 100 U/mL penicillin, (Sigma-Aldrich, St. Louis, MO, USA). The cells were incubated at 37 °C in a humidified incubator with 5% CO_2_ (Thermo Fisher Scientific, Waltham, MA, USA). To assess the impact of different CA3 concentrations on cell viability, the WST-1 assay was conducted following a previously described method [47]. The half-maximal inhibitory concentration IC_50_ values of CA3 were determined using a linear regression model, and these IC_50_ concentrations were used in further experiments. All experiments were conducted in triplicate for each cell line.

### 4.2. Protein Isolation and Proteomic Identification Through Nano-LC-MS/MS

Proteins were extracted from cells treated with CA3 at their respective IC_50_ concentrations and from untreated control cells. Each protein extraction was carried out in triplicate for all experimental conditions, using our previously described method [48]. After quantifying protein concentrations, equal amounts from triplicate samples within each group were pooled into single tubes.

Proteins from each group were digested before LC-MS/MS analysis using an in solution tryptic digestion kit (Thermo Fisher, Waltham, MA, USA), according to the manufacturer’s instructions. The obtained peptides were then analyzed via nLC-MS/MS [48]. A data-dependent acquisition strategy was employed, and the top ten precursor ions for MS/MS analysis within an MS scan range of 400–2000 m/z were selected. Proteome Discoverer SEQUEST software (version 2.2, Thermo Fisher Scientific, Waltham, MA, USA) was used to process the raw data. Protein identification was conducted against the Uniprot/SwissProt database to ensure precise and comprehensive characterization of the sample proteins.

### 4.3. Functional and Network Analysis of Proteomic Data

Differentially expressed proteins (DEPs) associated with treatment responses were systematically analyzed to explore their potential functional roles and interactions. Protein–protein interaction (PPI) networks were constructed to identify key regulatory proteins and clusters involved in the biological response to treatment. Additionally, pathway enrichment analysis was performed using the Kyoto Encyclopedia of Genes and Genomes (KEGG) and Gene Ontology (GO) databases to uncover significantly enriched biological processes, molecular functions, and cellular components associated with the DEPs. All computational analyses, including data visualization and interpretation, were conducted using ExpressAnalyst and SRPlot. All analyses were conducted with a *p* value < 0.05 and a gene count ≥ 2.

### 4.4. Protein Expression Profiling by Western Blot

Western blot analysis was performed to assess the expression levels of target proteins. A total of 25 μg of protein from each experimental group was separated by 12% SDS-PAGE and subsequently transferred onto nitrocellulose membranes following standard protocols [48]. Membranes were blocked with tris-buffered saline (TBS) containing nonfat powdered milk. Primary antibody incubation was carried out overnight at 4 °C using the following antibodies: anti-YAP (Cell Signaling Technology, Danvers, MA, USA, #4912), anti-LIG1 (Thermo Fisher Scientific, Waltham, MA, USA, MA1-23189), anti-PARP1 (Cell Signaling Technology, Danvers, MA, USA, #9532), anti-POLD3 (Thermo Fisher Scientific, Waltham, MA, USA, PA5-36951), anti-Beclin1 (Cell Signaling Technology, Danvers, MA, USA, #3495), anti-LC3 (Thermo Fisher Scientific, Waltham, MA, USA, BSM-51460M), and anti-GAPDH (Santa Cruz, Dallas, TX, USA, sc-81178). GAPDH served as an internal loading control to ensure equal protein loading. Following washes with TBS-T, membranes were incubated with an HRP-conjugated secondary antibody (Bio-Rad, Hercules, CA, USA). Protein bands were subsequently visualized using an enhanced chemiluminescence detection system (Bio-Rad, Hercules, CA, USA).

### 4.5. Flow Cytometric Assessment of Apoptosis and Cell Cycle

The apoptotic effects of YAP inhibition on MDA-MB231 and MCF7 cells were evaluated using Annexin V/propidium iodide (PI) flow cytometry analysis. Cells were seeded in 6-well culture plates at a density of 2 × 10^5^ cells/well and treated with CA3. The cells were then harvested by trypsin-EDTA, washed with PBS, and resuspended in binding buffer from the FITC Annexin V Apoptosis Detection Kit I (BD Biosciences, San Jose, CA, USA). Annexin V-FITC and PI were added to the cell suspension, incubated in the dark at room temperature, and analyzed using a FACSCalibur flow cytometer (BD Biosciences, San Jose, CA, USA) with CellQuest^TM^ software (BD Biosciences, San Jose, CA, USA, version 3.3).

To investigate the effects of CA3 on cell cycle progression, flow cytometric analysis was conducted using the CycleTEST™ PLUS DNA Reagent Kit (BD Biosciences, San Jose, CA, USA). Cells from each experimental condition were collected using trypsin and washed twice with a PBS and buffer solution. Then, 5 × 10^5^ cells per sample were sequentially treated with a trypsin buffer (Solution A) and incubated at room temperature for 10 min. A trypsin inhibitor and RNase buffer (Solution B) were then added, followed by another 10-min incubation. Finally, propidium iodide (Solution C) was added for DNA staining, and samples were incubated at 4 °C before analysis. Data acquisition was performed using a FACSCalibur flow cytometer (BD Biosciences, San Jose, CA, USA), and cell cycle distribution was analyzed using ModFit L^TM^ software (Verity Software House, Topsham, ME, USA, version 5.0). All experiments were performed in triplicate to ensure statistical reliability and reproducibility.

### 4.6. Statistical Analysis

The cytotoxicity, Annexin V apoptosis assay, and cell cycle progression data were analyzed using one-way analysis of variance (ANOVA) followed by Tukey’s post hoc test for multiple comparisons. The densitometric quantification of Western blot band intensities was performed using ImageJ software (National Institutes of Health, Bethesda, MD, USA, version 1.52a), with standard deviations calculated from at least three independent replicates. Statistical differences between experimental groups were assessed using Student’s *t*-test or one-way ANOVA, as appropriate. For nLC-MS/MS proteomic analysis, statistical evaluations were conducted using tools integrated within the Protein Discoverer software (version 2.2, Thermo Fisher Scientific, Waltham, MA, USA).

## 5. Conclusions

The interplay between the Hippo signaling pathway, DNA repair, and metabolism in breast cancer has been uncovered in this study. It was found that while luminal A-like MCF7 depends on YAP for survival, TNBC cells MDA-MB-23 resist YAP inhibition by boosting DNA repair and RNA metabolism, making them more aggressive. This suggests a potential vulnerability in TNBC, where simultaneous targeting of Hippo signaling and DNA repair pathways may improve treatment efficacy. Adding autophagy inhibitors could further enhance anti-tumor effects. In contrast, metabolic adaptation emerges as a key vulnerability in MCF7 cells, where defective DNA repair and reduced autophagy heighten their sensitivity to Hippo pathway modulation. Combining YAP/TAZ inhibitors with agents that block adaptive metabolic pathways could enhance treatment efficacy in luminal A-like breast cancers. Future research should explore these strategies in preclinical breast cancer models to develop better treatments.

## Data Availability

The datasets generated during the current study are available from the corresponding author upon reasonable request.

## Supplementary Materials

The following supporting information can be downloaded at https://www.mdpi.com/article/10.3390/ijms26093943/s1.
