# Supplementary material for: Targeting the Hippo Pathway in Breast Cancer: A Proteomic Analysis of Yes-Associated Protein Inhibition
Source: Int J Mol Sci. 2025 Apr 22;26(9):3943. doi: 10.3390/ijms26093943 (PMC12071972; doi:10.3390/ijms26093943)
Supplement: Supplementary file 1 [file ijms-26-03943-s001.zip › ijms-3560590-supplementary.pdf]

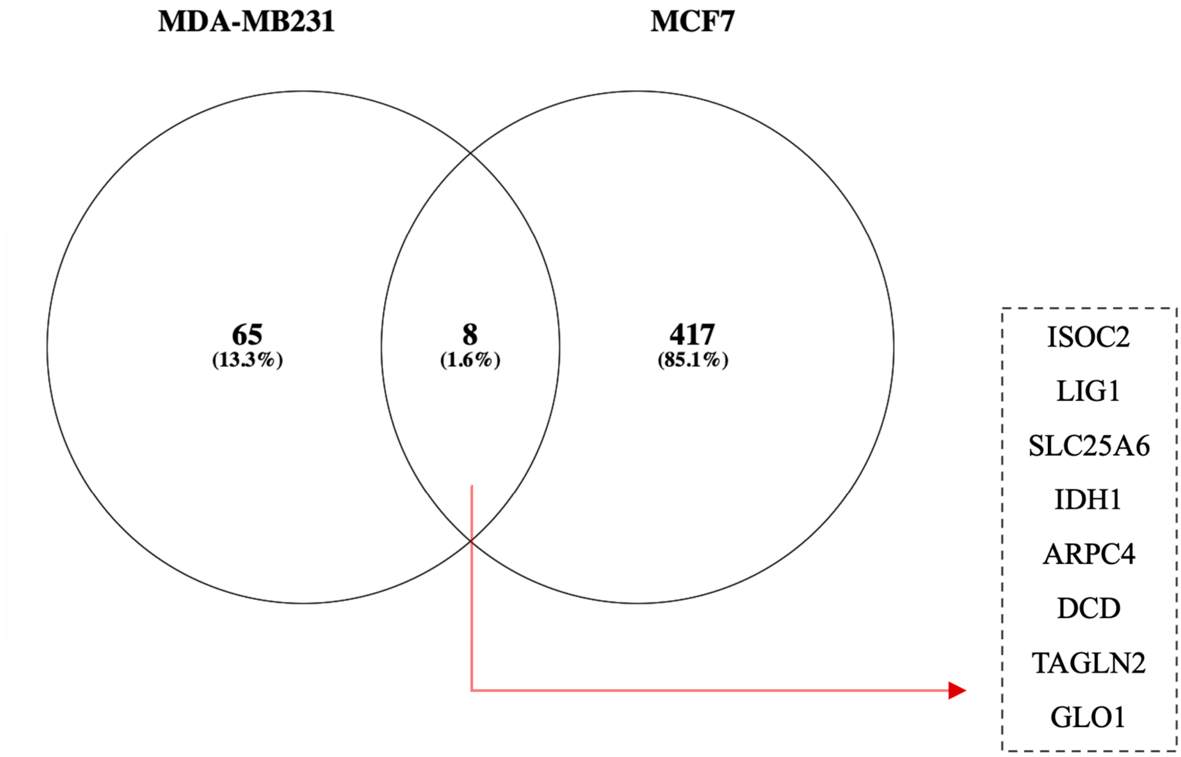


Figure S1: A Venn diagram showing the number of proteins identified by nLC-MS/MS and the common proteins that were regulated in both groups.
